# Electro-Encephalography: An Aid to Diagnosis

**Published:** 1940

**Authors:** W. Grey Walter

**Affiliations:** Physiologist to the Burden Neurological Institute, Bristol


### The Bristol Medico-Chirurgical Journal

*" Scire est nescire, nisi id me
Scire alius sciret."*

SPRING, 1940.

### ELECTRO-ENCEPHALOGRAPHY: AN AID TO DIAGNOSIS.

BY

W. Grey Walter, M.A.

*Physiologist to the Burden Neurological Institute, Bristol.*

The attitude of the medical practitioner to physiology is necessarily utilitarian and his familiarity with the various branches of the subject varies with their usefulness, not, as a cynic would suggest, with their simplicity. It is not surprising, therefore, that electro-physiology, though attracting great academic attention, is the least familiar of laboratory studies, for until recently the electro-cardiogram was the only application of the young and lively science whose *doyen* in this country is Professor Adrian of Cambridge. It is a commonplace of biology that every phase of living action is association with characteristic electrical changes, the "action potentials" of living cells. Why is it that the electro-cardiogram alone among action potentials has been impressed for service in the cause of medicine ? The reasons are chiefly technical. All the electrical signals of activity are small, and apparatus to record them must be either very sensitive and delicate or must incorporate elaborate devices to magnify these small electric discharges. Such apparatus is expensive and difficult to keep in order, so that except in the case of the heart, which happens to provide one of the largest bio-electric potentials, the electrically interesting parts of the body have remained inaccessible except to those undismayed by such words as "grid leak" and "high frequency pentode."

In the last few years, however, a branch of electro-physiology has developed a definite degree of clinical importance, although it involves equipment even more impressive than that to be found in most physiological laboratories. This is electro-encephalography, which is beginning to do for the brain what electro-cardiography does for the heart.

It has been known for many years that the exposed brain of an animal produces electrical discharges on stimulation of a sense organ, and also that it frequently develops spontaneous rhythmic discharges independently of sensory stimulation. But the human brain was not explored electrically until recently, partly because it was not considered justifiable to prolong a cranial operation merely to this end : and it was not until 1929 that Berger of Jena showed that the spontaneous electrical activity of the human brain could be detected by connecting electrodes on the scalp to sufficiently sensitive recording apparatus. Berger claimed that when his subjects were resting quietly he could detect rhythmic electrical waves at a frequency of about 10 per second. These were very small, about one-hundredth the size of those of the electro-cardiogram, and were diminished by any sort of mental or visual activity. Berger called these "alpha waves." He also thought he detected faster "beta waves" of more variable behaviour. This discovery was neglected until Adrian surprised himself by confirming it in 1934. Adrian and his colleagues went further and localized the origin of these waves to the occipital visual association cortex, and they emphasized that they were waves of rest, not action, disappearing when the eyes were opened or when the mind was active. They may be considered as a sign that the visual association areas are "marking time." These "inaction potentials," the alpha waves, are the only definite and universally accepted normal electrical discharge from the human brain seen when electrodes are placed on the scalp. There are other normal discharges, but they are too small to be seen unless electrodes are placed directly on the cortex exposed at operations.

Being a Professor of Psychiatry, Berger soon turned his attention to the pathological, but his results were clouded by controversial and sometimes incoherent interpretation; nor was his equipment adequate for this purpose. Working originally on the normal electro-encephalogram Walter happened in 1936 to take records from a few patients suspected of having cerebral tumours. He found that these records showed slow waves entirely unlike the normal ones, and by using three recording systems simultaneously he was able to locate the origin of these slow waves, which he called "delta waves." The patients whose "delta foci" were located in this way were subsequently found to have tumours in or very near to the region indicated by electro-encephalography. The series begun with these few chance cases has now grown to several hundred, and the following findings would be agreed to by the numerous other workers who have confirmed Walter's work :—

1. The great majority of tumours which affect the cortex either directly by infiltration or indirectly by other means produce in it a condition in which a delta discharge occurs.

2. The new growth itself is entirely inactive electrically.

3. When the intercranial pressure is grossly raised the whole cortex may show a delta discharge. If the pressure can be lowered by injection of hypertonic solutions the general discharge will diminish, unmasking the local abnormality.

4. The greater and more acute the damage the slower are the delta waves. Benign tumours may give rise to a discharge almost as rapid as the normal alpha rhythm. A very benign, long-standing tumour may give scarcely any sign of its existence. For this reason a *normal* electro-encephalogram must be regarded as inconclusive.

5. Tumours below the cerebral hemispheres cannot be localized, though their indirect effects can sometimes be observed.

The above observations apply to every "space-occupying lesion," such as a cerebral abscess or a hæmatoma. It is important to note that in a case of hæmatoma the abnormal discharge is produced only during the first few weeks of its existence, and the same applies to trauma.

The delta discharge is a non-specific sign of cerebral distress. The cells may go on to a terminal stage of atrophy or recover entirely. In all conditions but one, the time for which a given area will produce a delta discharge is strictly limited. That one condition is idiopathic epilepsy. Golla, Graham and Walter showed in 1937 that about half of a group of several hundred "persons complaining of fits" showed a continuous resting abnormality in the electro-encephalogram. This resembles in some ways the tumour records, but the discharge is more rhythmic. Here, again, the results have been confirmed by many observers and may be summarized as follows:—

1. About 50 per cent. of epileptic patients show a continuous electro-encephalographic abnormality.

2. In a patient complaining chiefly of *grand mal* attacks, the focus of the abnormality is in the region of the superior frontal gyrus on either or both sides.

3. In a patient with only minor attacks the focus is often post-central.

4. During an attack the abnormality increases enormously in area and magnitude. A major attack is characterized by a rapid discharge, a minor one by a mixed fast and slow "spike and wave" discharge, a psychomotor or "equivalent" by a smooth, slow discharge.

5. In most epileptic patients whose resting electro-encephalograms are normal, a clear abnormality can be evoked by a few minutes voluntary over-breathing. In about 10 per cent. of such cases a fit will also be induced.

6. If the patients are divided into age groups, those above forty will contain few with abnormal electro-encephalograms, those below twenty a high proportion. By using over-breathing nearly all epileptic children are found to have abnormal electro-encephalograms, and a certain number whose complaint was merely bad behaviour also show abnormalities.

7. The resting abnormality is not greatly affected by drugs even when these succeed in controlling the fits: sedation seems merely to discourage the abnormality from spreading over a wider area.

8. Cases of true traumatic epilepsy do not show a resting abnormality, although during a fit the record is the same as in an idiopathic case. (Also in fits induced with Cardiazol.)

In general, electro-encephalography provides a real positive test whereby, in young persons at least, true essential epilepsy may be distinguished from hysterical or functional conditions, although as with cerebral tumours a normal or negative finding does not give the patient a clean bill.

There is no space to discuss the brilliant though somewhat oblique light which these discoveries have thrown on the ancient problem of epilepsy. As may be imagined, it has revealed enormous gaps in our fundamental knowledge and cast deep shadows on some of our most cherished axioms. That electrical changes are closely associated with the fit-producing mechanism seems extremely likely ; indeed, electrical stimulation is now being used to induce convulsions in the treatment of schizophrenia. Perhaps it is not too optimistic to hope that by combining the study of these artificial fits with the electrical data concerning epilepsy a new epoch in the study of convulsive disorders may be opened.

In application, electro-encephalography can never displace or supplant the conventional clinical methods. In cases of cerebral tumour it can supply the same sort of information as air-replacement with the disadvantage that sub-cerebral lesions cannot be accurately detected, and the great advantage that it causes absolutely no pain or danger to the patient. In epilepsy the electrical supplement to the clinical examination and history is of unique value. True, it is still only of academic interest to know from what part of the brain a fit starts, but the immediate painless result eliminates the long and often fruitless wait before a fit is observed. Moreover, a positive finding in a young person suffering from a " behaviour disorder " can greatly simplify the task of the psychiatrist.

In some cases interpretation of the records is difficult and certain points of detail are still the subject of controversy, as is also the case in electro-cardiography, but it is safe to say that the discovery of slow delta waves in any patient suspected of suffering from a cerebral tumour, cerebral abscess, intracranial hæmatoma, early head injury, or essential epilepsy is reliable evidence of an organic basis for the trouble ; in most cases the position of the lesion can be indicated and in some its nature may be suggested. The method is still too complicated to be used by anyone but an electrical expert, but in the course of time it may be considerably simplified for clinical purposes.

